# Severe acute respiratory syndrome coronavirus 2 (SARS-CoV-2): a review

**DOI:** 10.1186/s12943-020-01218-1

**Published:** 2020-06-02

**Authors:** Wei Feng, Wei Zong, Feng Wang, Shaoqing Ju

**Affiliations:** Department of Laboratory Medicine, Affiliated Hospital of Nantong University, No 20, Xisi Road, Nantong, 226001 China

**Keywords:** Coronavirus, SARS-CoV-2, Epidemic, Virus detection

## Abstract

In recent years, the prevalence and spread of coronavirus has had a huge impact on global public health. Due to the incomplete understanding of the pathogenic mechanism of the virus, it is difficult for humans to fight against the virus quickly and effectively once the outbreak occurs. In early 2020, a novel coronavirus was discovered in Wuhan, China. Soon after, similar cases were found in other countries around the world, and the number of infected people increased rapidly. So far, the global cumulative number of infected people has exceeded 3 million, and more than 200,000 people have died, which has had a huge impact on global human health and economic development. Every outbreak of disease makes a deep impression on mankind. Herein, we summarize the virology, epidemiology, clinical manifestations, diagnosis, treatment and prevention of SARS-CoV-2, and hope that countries can control the outbreak as soon as possible to minimize the loss.

## Background

It is known to all that since the twenty-first century, there have been three human pathogenic coronavirus outbreaks, which have caused global transmission, bringing huge challenges to global public health and economic development [1]. They are the Severe Acute Respiratory Syndrome coronavirus (SARS-CoV) in 2003 [2], the Middle East Respiratory Syndrome coronavirus (MERS-CoV) in 2012 [3], and the new coronavirus (Severe Acute Respiratory Syndrome coronavirus2, SARS-CoV-2) in 2019 [4]. At present, there is no specific treatment plan.

In early 2020, a case of novel coronavirus was confirmed in Wuhan, China [4]. Within a short period of time, the number of confirmed cases was increasing, and those infected could develop fever, cough, and even severe respiratory syndrome [5], which quickly drew the attention of the Chinese government [6, 7]. Researchers found that 27 of the 41 initially confirmed cases had had direct contact with a local seafood market in Wuhan, initially assuming that the new coronavirus might have come from wild animals sold in the market [8]. Gao et al. collected alveolar lavage fluid from three infected patients and successfully isolated the new coronavirus [9]. Electron microscopy has showed that the virus has an envelop, the particles are round or oval with the diameter of about 60–140 nm [4]. Whole genome sequencing analysis has showed that the virus belongs to a new type of coronavirus of the β genus [4]. In addition, Shi et al. obtained almost identical genome-wide sequences in virus samples from 5 patients. The sequence of the new coronavirus was 79.5% similar to SARS-CoV and 96% similar to the coronavirus carried by bats, which might be the potential source of infection [10]. Another study found that the receptor binding domain of SARS-CoV-2 was the closest to the coronavirus carried by pangolins. Thus, the origin of SARS-CoV-2 remains to be determined [11].

**Fig. 1:**
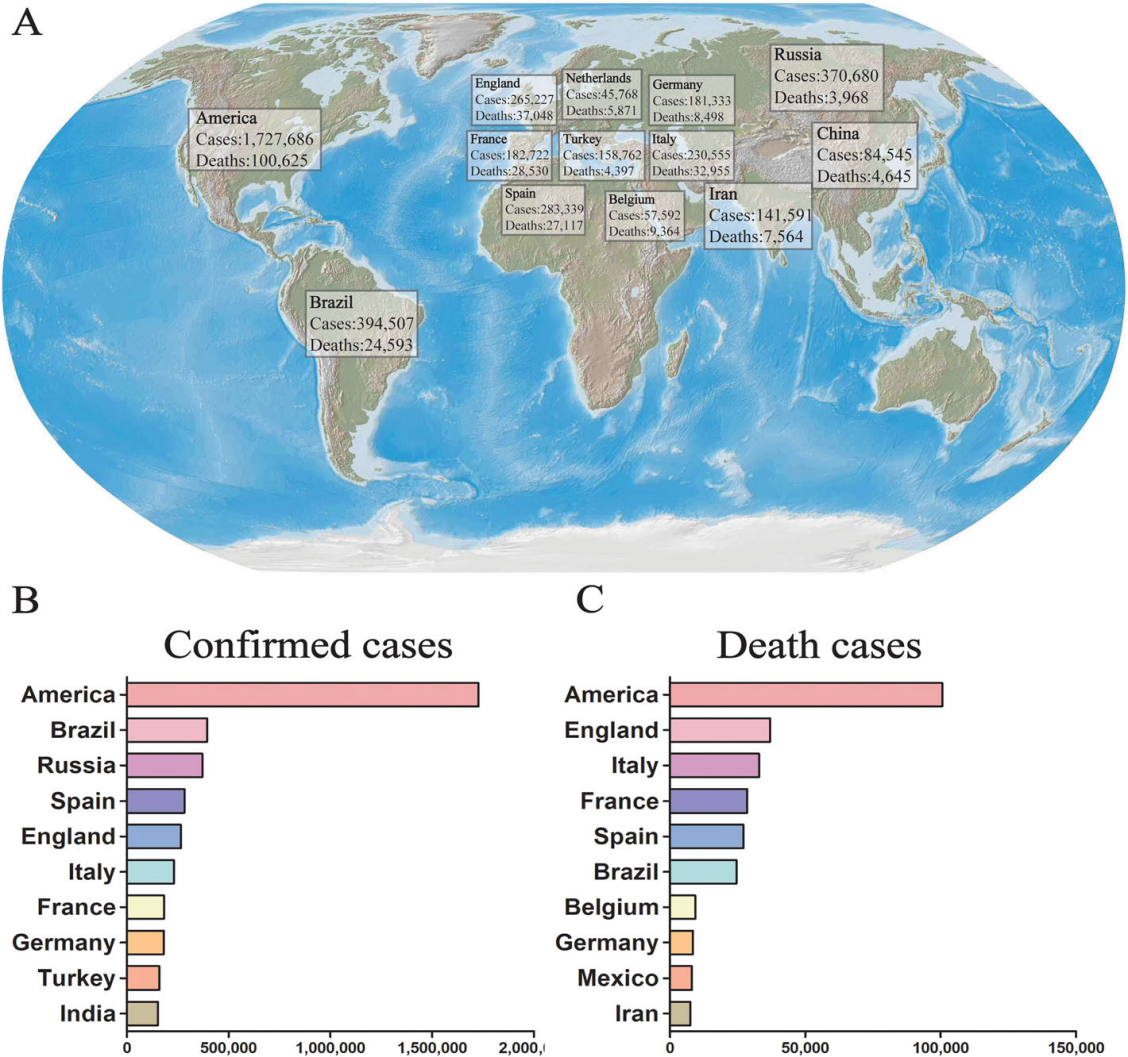
The latest data on SARS-Cov-2 on 27 May 2020. **a** SARS-Cov-2 mainly affects countries; **b** top 10 confirmed SARS-Cov-2 cases; **c** top 10 SARS-Cov-2 deaths

The World Health Organization (WHO) officially declared SARS-CoV-2 to be a public health emergency of international concern on January 31, due to its rapid spread. Furthermore, the WHO declared SARS-CoV-2 to be a global pandemic on March 11, 2020 [12]. The last time that the WHO declared a pandemic was H1N1 in 2009, which affected more than 70 countries and infected more than 60 million people in the United States alone [13]. As of May 27, 2020, SARS-CoV-2 had a total of 5,715,077 confirmed cases worldwide, including 352,912 deaths. Therefore, it can be seen that it has strong transmission and pathogenicity (Fig. 1).

This review mainly investigates the current status of the epidemic, provides information of SARS-CoV-2 on virology, clinical features, epidemiology, diagnosis, treatment, prevention, and the role of internet new media, highlights the major issues that still need to be studied and addressed.

## Virology

Coronavirus is the longest known RNA virus, with a length of 27–32 kb [14]. In 2014, the International Committee on Taxonomy of Viruses (ICTV) divided coronaviruses into four genera, α, β, γ, δ. Of the four coronavirus genera, seven have been found to be pathogenic to humans, α: HCoV-229E, HCoV-NL63; β: HCoV-OC43, HCoV-HKU1, SARS-CoV, MERS-CoV. The newly discovered SARS-CoV-2 also belongs to the β genus [9, 15].

**Fig. 2:**
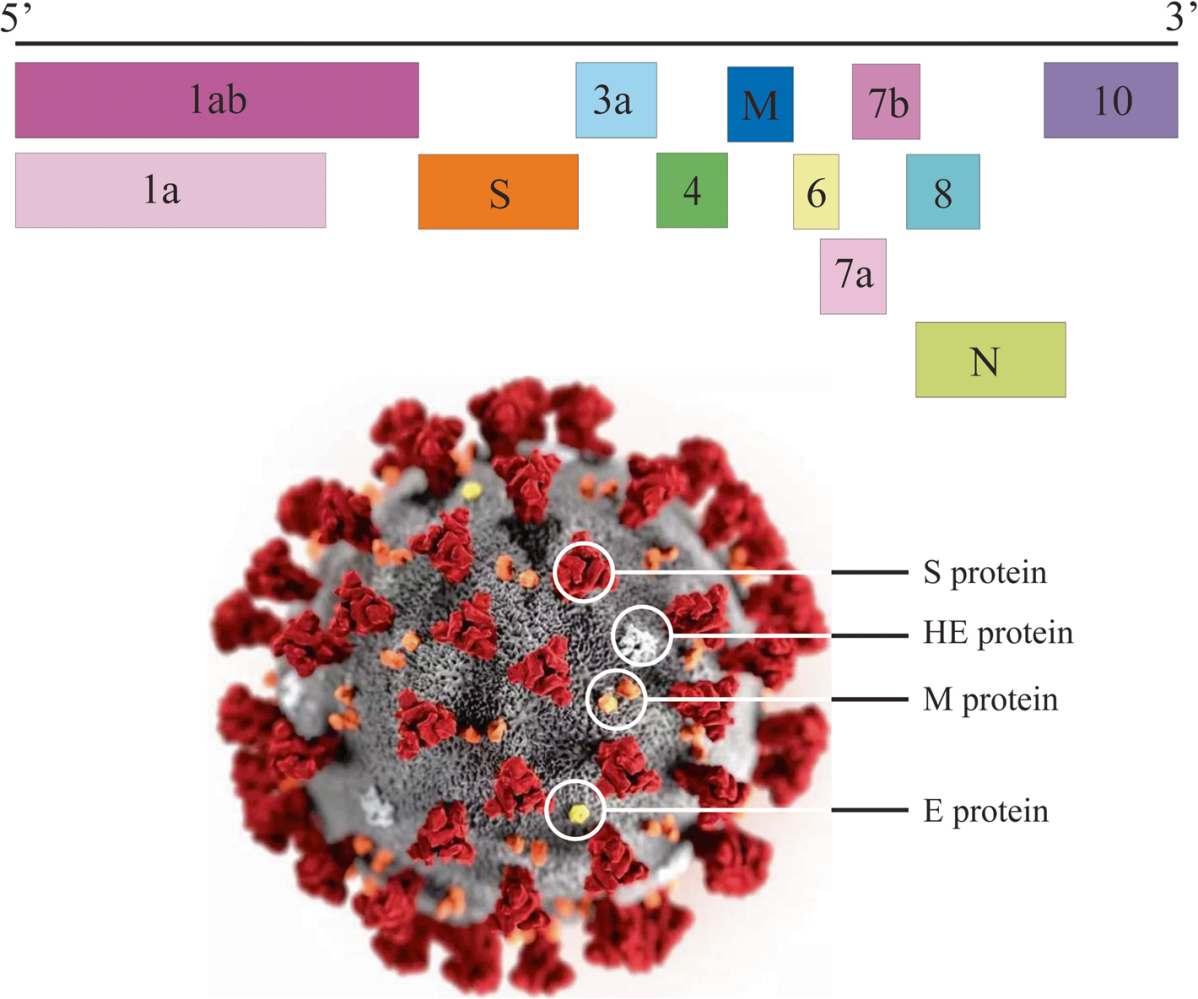
SARS-Cov-2 genome organisation and structure

**Fig. 3:**
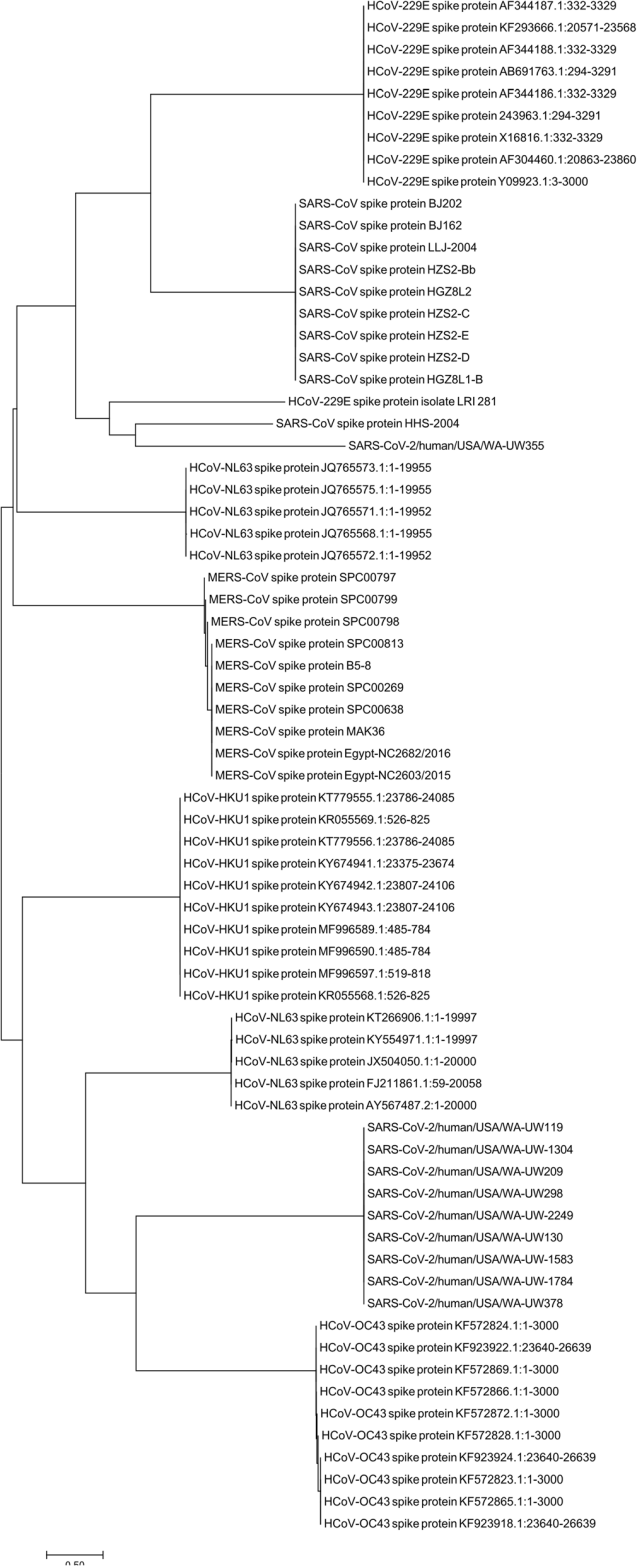
Phylogenetic tree of human pathogenic coronavirus S protein

The reason that coronavirus can cause human pandemic is closely related to its special structure [16, 17]. The sequencing results of SARS-CoV-2 genome sequence showed that its genome contains about 29 kb bases and 11 protein-coding regions (Fig. 2), including 1ab, 1a, S, 3a, 4, M, 6, 7a, 7b, 8, N, 10 [16]. The entry of SARS-CoV-2 into host cells was mediated mainly by the use of spike protein (S) [18]. Therefore, we performed phylogenetic analysis of S protein of the human pathogenic coronavirus (Fig. 3). S protein is a prominent trimer structure on the surface of the virus, containing two functional subunits S1 and S2, where S1 is responsible for binding to host cell surface receptors, and S2 is responsible for membrane fusion between the virus and the cell. There is a furan cleavage site at the boundary between S1 and S2 subunits, which is mutated during virus invasion, thus distinguishing SARS-CoV-2 from SARS-CoV [16]. Veesler et al. showed that SARS-CoV-2 entered cells using S protein by utilizing the angiotensin-converting enzyme 2 (ACE2) receptor on the surface of host cells, which is widely present in human respiratory and intestinal mucosal epithelial cells, enabling SARS-CoV-2 to infect humans and cause transmission in humans, which is similar to the transmission mechanism of SARS-CoV [16, 19]. In addition, studies have shown that the expression level of ACE2 gene in the lungs of smokers is significantly higher than that of non-smokers, and smokers are more likely to be infected with SARS-CoV-2 [20]. These findings provide an important theoretical basis for drug development and epidemic prevention and control. In addition, an analysis of the SARS-CoV-2 genomic phylogenetic network revealed three types of variation, which the researchers tentatively named A/B/C, where A is the ancestor type, A and C mainly exist in Europe and the United States, while B mainly exists in east Asia, providing a new idea for the origin of SARS-CoV-2 [21].

## Clinical features

### The incubation period

The incubation period, it is to point to from causative agent invader airframe or produce action to airframe commonly, arrive airframe begins to appear reaction or clinical symptom when arrive. The incubation period of different infectious diseases is different, mainly related to the number of pathogens, pathogenicity, feculence and the autoimmunity of the infected person [22]. Determining the incubation period of infectious diseases is helpful for researchers to determine the time of infection of an person, track the source of infection, and determine the route of transmission [23, 24]. It can also provide an important theoretical basis for the air defense time of prevention and quarantine of infectious diseases. On January 31, 2020, the U.S. Centers for Disease Control and Prevention (CDC) announced that all residents returning from the outbreak would be subject to a mandatory 14-day quarantine.

Through investigation and analysis of 88 patients diagnosed with SARS-CoV-2 in the early stage, Backer et al. preliminarily proved that the average incubation period of the disease was 6.4 days (95% CI, 5.6 to 7.7 days) [25]. Another study on the incubation period of SARS-CoV-2 showed that the incubation period of the infectious disease ranged from 2 days to 14 days [26]. The latest study analyzed 181 cases of SARS-CoV-2 infectious using the log-normal model and predicted that the patients would show clinical manifestations within 12 days [23]. The above research indicates that the average incubation period of SARS-CoV-2 is about 14 days, but it is possible to extend the isolation observation period appropriately if necessary.

### Adult performance

The clinical symptoms of patients infected with SARS-CoV-2 are mainly fever, fatigue and cough, but more than 50% of the infected patients even have no obvious symptoms, which is easy to cause missed diagnosis and misdiagnosis [27, 28]. In addition to respiratory symptoms, some people who are infected also experience digestive symptoms such as loss of appetite, nausea and vomiting [29]. Some severe cases can progress to acute respiratory distress syndrome or even multi-organ failure (ECMO plays an important role in the treatment of critically ill patients) [30, 31]. Diarrhea, a common symptom of SARS-CoV-2 infection, also accounts for a high proportion of SARS-CoV-2 infections [32, 33]. In addition, the total number of white blood cells in adult infected patients is normal or slightly decreased in general laboratory examination, in which the decline of lymphocytes is more obvious, C-reactive protein (CRP) can have a slight increase, and there is a slight increase in erythrocyte sedimentation rate (ESR) [34]. In obese patients, aspartate aminotransferase, LDL cholesterol and lymphocyte counts decreased significantly [35]. Meanwhile, a retrospective study found statistically significant reductions in eosinophil levels in patients with poor prognosis [36]. Imaging finding suggested that most of the infected patients had pneumonia. Chest CT is usually characterized by ground-glass opacity (GGO). Progressive chest CT indicated increased lesions, the accumulative range of lesions was expanded, and in severe cases, diffuse consolidation of both lungs presented as “white lung” [37, 38]. So far, most of those infected have mild symptoms and a good prognosis, but the mortality is as high as 17% for the elderly or those with chronic disease [39]. According to the statistical analysis of the current infected data, the infected age is mainly between 40 and 60 years old, among which the male patients are slightly more than the female patients, the infected children are rare, and most of the families of infected children have other family members infected [40]. In addition to the typical clinical manifestations of the respiratory and digestive systems, many patients were treated for obvious skin manifestations (mainly red rash on the back of trunk, urticaria, etc.). Therefore, it is necessary for our clinicians to find such patients in time to avoid misdiagnosis and missed diagnosis [41, 42].

### Children performance

Although the current statistical analysis shows that the infection rate of children is low, it does not mean that children are not susceptible to disease, perhaps because children have less contact with the outside environment and most families pay more attention to the health protection of children, which need to be taken into account. Some studies have found that the average incubation period of children is longer than that of adults, the clinical manifestations of infected children are relatively mild, cough and fever are also common manifestations, symptoms tend to subside, slightly infected people or even no symptoms, just positive nucleic acid test [43]. More than 80% of infected children have detectable viral nucleic acids in their faeces [44]. One study found that of the 10 children infected outside the Wuhan, 8 had direct contact with adults with a history of travel to the city. The symptoms of infection in children are relatively mild and not easy to be found, but the imaging examination often shows patchy infiltration, if not timely treatment, children’s growth and development is seriously affected [27, 45]. Therefore, children who may contact with high-risk groups should be closely monitored and isolated for early detection and intervention [45, 46]. Since the symptoms of infected children are mild, treatment with large doses of antiviral drugs or empirical antibiotics is not necessary, unless the condition is severe and no other specific treatment options are available [47].

## Epidemiology

### Source of infection

Studies have shown that coronavirus α and β originated in bats, and the other two viruses originated in birds [1]. A comparative analysis of genome sequences showed that SARS-CoV-2 was 96% similar to coronavirus from bats, and bats were likely the natural host of SARS-CoV-2 [10, 48]. However, there may be one or more unkonwn intemediate hosts between bats and humans, just as the intermediate host of SARS-CoV is civet cat and the intermediate host of MERS is dromedary camel [49, 50].

Most of the original SARS-CoV-2 infections came from a local seafood market in Wuhan [51]. The internal environment of the market was similar to the place where SARS-CoV was discovered in 2003 [52]. The wet and dirty environment provides favorable conditions for the virus to survive and reproduce. In addition, a variety of seafood was sold in the market, and there was also illegal trafficking of wild animals, the pathogen was likely to be one of the intermediate hosts or some wild animals [53]. According to the latest research, the virus strain isolated in pangolins is 99% similar to SARS-CoV-2 in sequence [54]. It is suspected that pangolins may be the potential intermediate host of the virus. Pangolins were also sold in the market [55]. It was likely that the first infected people ate the infected pangolins, causing human-to-human transmission. After the outbreak, the local government moved quickly to close the market and conducted a thorough disinfection. Of course, pangolins may not be the only intermediate host, as coronaviruses have been found in many animals, such as camels, pigs, goats, and pangolins. With further research, it is believed that more potential intermediate hosts will be found [56]. In addition to wild animals, the researchers found that chickens, ducks, pigs and dogs were less susceptible to SARS-CoV-2, while cats were more susceptible to SARS-CoV-2, providing important information for future animal management [57]. After the outbreak, many regions in China have introduced relevant laws and regulations, and some animals have been put on the fasting list.

### Route of transmission

At present, the pathogenic mechanism of SARS-CoV-2 is still little studied, and its transmission route and transmission mode are also greatly uncertain due to virus mutation and other factors. Epidemiological data show that SARS-CoV-2 has a strong infectious capacity and is highly infectious. The known transmission route is mainly based on the history of the infected person, and the sample of SARS-CoV-2 can be detected to make a guess. The presence of SARS-CoV-2 was found in nasopharyngeal swab specimens of the infected person, and the cases of cluster infection all suggested that airborne droplet transmission was the possible main transmission route [58–60]. Confined space, poor ventilation and so on are important causes of cluster infection [61]. To this end, on January 23, 2020, the Chinese government imposed a “city closure” on Wuhan and travel restrictions on other cities to minimize unnecessary crowds. Currently, disseminated by respiratory droplets and direct contact is clear, a study collected in different regions of the hospital air SARS-CoV-2 testing, found in the region of the medical staff and room toilet space existing in the small area is relatively high concentrations of the virus nucleic acid, but whether these aerosol form of the virus has spread ability is not yet clear, these need to pay attention to the prevention and control of hospital [62, 63]. SARS-CoV-2 RNA was also detected in blood samples from infected patients [5, 64, 65]. The real-time fluoresence quantitative PCR showed that the PCR cycle threshold was 35.1, indicating that there was a low concentration of viral nucleic acid in the blood samples, and blood transmission might also be a potential transmission route of SARS-CoV-2 [66]. It also increases the difficulty of blood donation in blood centers and blood banks. The high expression of ACE2 receptor in the gastrointestinal tract indicates that SARS-CoV-2 can use ACE2 receptor in the gastrointestinal tract to enter the human body, so the fecal-oral pathway may be a potential transmission route of SARS-CoV-2 [67]. It is reported that the Chinese center for disease control and prevention isolated the SARS-CoV-2 strain from stool samples from a confirmed patient in Heilongjiang Province, indicating that SARS-CoV-2 could survive in feces. A similar study found low levels of SARS-CoV-2 in the feces of some infected children [44]. In addition, the government of Paris detected a novel coronavirus in a non-potable water system and an active novel coronavirus in sewage in Massachusetts, USA. These studies have shown that a novel coronavirus can survive in water and be transmitted using water. Therefore, if the water is infected by the virus, even with the current stringent control measures, there is still a high risk of human infection. A newborn born during the outbreak at a local children’s hospital in Wuhan was reported to have been diagnosed with SARS-CoV-2 infection 30 h after birth, and it was considered that SARS-CoV-2 might also cause an epidemic through vertical transmission [68]. However, another study did not find the presence of SARS-CoV-2 in the samples of amniotic fluid, breast milk and other body fluids of 9 pregnant women infected with SARS-CoV-2 [69]. The newborns were not infected, and no evidence of vertical transmission of pregnancy was found, possibly due to the small sample size. For health workers, the possibility of vertical transmission still exists and needs to be taken seriously in the process of disease control [70]. In addition, there have been reports of infection in laboratory specimens, and human conjunctival epithelium is susceptible to infection by infectious aerosols [71].

### Infected people

An important indicator to measure the ability of the disease to spread: Basic reproduction number (R0), refers to the average number of infectious diseases that can be transmitted to others without the need for external intervention [72]. The critical value is 1. The greater the R0 value, the more difficult to control the epidemic. With the participation of all countries in the world, the R0 value is also changing. Initially, Zhao et al. estimated that the initial R0 value of SARS-CoV-2 was between 3.5 and 5.5, slightly higher than SARS-CoV. The latest study shows that R0 is around 2.68, roughly the same as the R0 reported by the WHO and the Chinese CDC [73, 74]. At present, SARS-CoV-2 is spreading rapidly all over the world, with the cumulative number of infected people excedding 160,000. If no corresponding measures are taken, the epidemic situation will worsen. As a “global village” such a big family, the global countries should unite and work together to control the R0 value below 1 [75]. A retrospective study of patients infected with novel coronavirus in a local hospital in Wuhan found that elderly men, patients with hypertension and heart disease were more likely to suffer from serious complications, with a poor survival prognosis and a high mortality rate, which were required close attention [76].

## Diagnosis

### Laboratory diagnosis

The diagnosis of infectious diseases depends on the identification of the pathogen [77, 78]. The identification of pathogens mainly includes “virus isolation” and “nucleic acid detection”, but because “virus isolation” requires a high level of biosafety in the laboratory, it is mainly used in scientific research. Clinical diagnosis of SARS-CoV-2 mainly relies on “nucleic acid testing”. According to the transmission characteristics of SARS-CoV-2, nasopharyngeal swabs, sputum, respiratory secretions, alveolar lavage fluid, blood, feces and other samples of the infected person can be used as the source of samples for viral nucleic acid detection [79]. In a study, by detecting the nucleic acid content of novel coronavirus in sputum samples of 92 infected patients, it was found that the virus content in sputum samples of severely infected patients was higher than that of mildly infected patients, among which 11 patients with aggravated illness had higher nucleic acid content of novel coronavirus in sputum samples [80]. In addition to detecting viral nucleic acids, antibody detection in serum of healthy people is a relatively fast screening method. Studies have shown that IgG and IgM can be detected in the second week after SARS-CoV-2 infection [81]. At present, SARS-CoV-2 nucleic acid detection kit approved by China national medical products adminstration mainly uses real-time fluorescence quantitative PCR method to detect specific genes of viral nucleic acid [82]. The detection kits of real-time fluorescence quantitative PCR are mainly divided into two categories: double real-time fluorescence quantitative PCR and triple real-time fluorescence quantitative PCR, both using Taqman probe method. The target genes detected by double method were mainly 1ab and N gene, while those detected by triple method were 1ab, N and E gene. In addition, viral genome sequencing can also identify the sequence and function of SARS-CoV-2. This method is better than the real-time fluorescence quantitative PCR method for the detection of early samples with low virus content, but it is also less applied due to the lack of sequencing equipment and bioinformatics analysts. Therefore, real-time fluorescence quantitative PCR is the main method to detect viral nucleic acid. As a designated hospital for local nucleic acid testing, the department undertakes the testing of most suspicious samples in the region and surrounding areas. In order to ensure reliable test results, we have developed a set of quality control procedures, hoping to provide assistance for nucleic acid testing.

#### Specimen quality assessment

The quality of samples determines the sensitivity and specificity of viral nucleic acid detection. The virus detection rate of bronchus/alveolar lavage fluid is relatively high due to the relatively high viral load in the respiratory tract specimens of infected persons, but sampling is difficult to get. Routine clinical collection of nasopharyngeal swabs, blood, stool and other samples, but the collection of these samples is susceptible to sampling materials, sampling sites, sampling time and sampling personnel’s operation. Therefore, it is recommended to test negative specimens for patients with suspicious clinical symptoms multiple times, take samples from multiple sites, and conduct standard sampling by skilled personnel.

#### Evaluation of test results

Due to the sudden outbreak of the epidemic, there are not enough clinical samples of the detection kits developed by various companies or scientific research insitutions for complete performance confirmation. Therefore, the stability and reliability of various nucleic acid detection kits need to be further verified. Here, it is recommended to use the detection kit containing the control, and it should match with the laboratory equipment, and extract virus samples in strict accordance with the standard operating procedures of the kit, and set up detection procedures. The detection personnel should also analyze the situation of the whole amplification curve and the individual amplification curve after amplification, and the amplification curve is required to present a single peak specific smooth curve, followed by adjusting the detection procedure according to the dissolution curve. In addition, a reasonable indoor quality control scheme is of vital importance. We suggest that one weak positive quality control and three negative quality controls should be set up for each batch of test. All three negative quality controls should be negative, and weak positive quality control should be positive, which is considered as the control of this batch of test. Otherwise, it is out of control and cannot issue a test report. If necessary, it should be retested. For positive results, we should ensure that the fluorescence quantitative PCR results of at least two targets (1ba/N/E) in the test are positive. When only one target is positive, rechecking and even resampling should be requested as much as possible. For the negative results, the infection cannot be completely excluded, and comprehensive analysis of the patient’s symptoms, medical history and imaging findings is also needed [83, 84]. Standard and reasonable test report is very important for clinicians to make correct judgment. With the spread of SARS-CoV-2, countries all over the world need rapid and effective methods to detect their own people. A rapid detection method of CRISPR-cas12 was found, which was mainly used to detected SARS-CoV-2 in respiratory throat swab, with a sensitivity of up to 95% and specificity of 100% [31]. There are similar colloidal gold immunochromatography, side flow immunoassay, which can quickly and sensitively detect specific antibodies in the serum [85].

### Imaging presentation

In addition to laboratory tests, imaging findings are critical for disease diagnosis. Routine X-ray examination is convenient and quick. However, because the overlapping images will affect the observation of doctors on the lesions, and the sensitivity and specificity are low, it is easy to miss diagnosis, which has not played an obvious role in this outbreak. CT examination is another important imaging examination for respiratory diseases and plays an important role in the diagnosis of SARS-CoV-2 pneumonia [86]. Patients infected with SARS-CoV-2 can be divided into three stages according to their condition. The early CT manifestations of the lesion were mainly local lesions, patchy, segmental distribution, and the lesions were mostly seen in the outer 1/3 lung field and subpleural. In the progressive stage, CT mainly presents increased lesions with expanded scope, involving multiple pulmonary lobes, with most of the lower lobes. In the critical stage, CT mainly presents diffuse lesions, lung consolidation, distorted structure, and “white lung” in severe cases [37, 38]. Imaging findings of diseases also played a crucial role in early diagnosis, which should be paid more attention in the case of epidemic [87, 88].

## Therapy

In addition to early detection and early diagnosis of the disease, early treatment is crucial to the prognosis of the disease. Since the outbreak, many research institutions around the world have been conducting clinical trials, using existing potential drugs for in vivo and in vitro research. At present, there are mainly antiviral drugs, anti-inflammatory drugs, and some drugs have achieved significant effects, but it is still some time before the treatment of COVID-19 patients [89, 90]. Studies have shown that ACE2 is the receptor for SARS-CoV-2 to enter cells, and ACE2 is present in large quantities in human lung and small intestinal epithelial cells, which is essential for the spread of the epidemic. Similarly, ACE2 is a potential therapeutic target [91, 92]. It has been found that recombinant human soluble ACE2 can inhibit the growth of SARS-CoV-2 and significantly block the early stage infection of novel coronavirus, which is an important discovery for the treatment of patients with new crown disease [93]. In addition, the use of ACE inhibitors or angiotensin receptor blockers (ARBs) can provide protection to the cardiovascular and renal systems of COVID-19 patients [94]. There are many similar therapies that inhibit the action mechanism of S protein and ACE2 [95]. A combination of broad-spectrum antiviral drugs, lopinavir and ritonavir has been recommended for clinical trial in the latest guidelines [96]. In addition, Remdesivir, also a broad-spectrum anti-coronavirus drug, has been shown to inhibit SARS-CoV-2 cell infection in vitro [89]. The first SARS-CoV-2 patient in the United States showed a marked improvement in symptoms after being treated with RDV, which opened the possibility for further clinical application [97]. Clinical trials of the drug have also been carried out in China, which has a large number of infected people. Favipiravir was approved for the treatment of influenza virus infection in 2014. Through the clinical comparison of 200 COVID-19 patients, it was found that the pneumonia symptoms of the patients receiving favipiravir were significantly improved, with no obvious side effects [98]. With the increasing number of studies on confirmed cases, some drugs with immunoregulatory and anti-inflammatory effects have also been used in the treatment of COVID-19 patients. Studies have found that the hypercoagulable state of blood is related to the severity of the disease. An anticoagulant drug dipyridamole (DIP) can inhibit the replication of novel coronavirus, significantly improve the condition of severe patients, and play a positive role in the treatment of patients [63]. Chloroquine, originally used to treat malaria, was found to inhibit the activity of the SARS virus in vitro during the SARS epidemic, mainly by altering the glycosylation of the viral receptor ACE2, thereby affecting the virus to invade cells. Recent studies have shown that the combined application of ridsivir and chloroquine can effectively inhibit the in vitro activity of SARS-CoV-2 [99]. In addition, chloroquine also has immunomodulatory effects, can inhibit the emergence and progression of viral diseases [100]. Abidol has a broad spectrum antiviral effect on respiratory virus and can also inhibit the activity of SARS-CoV-2 in vitro [101]. Currently, the treatment of new coronavirus pneumonia by abidol has entered into phase 4 clinical trials, and is likely to be applied in treatment in the future. Many studies have shown that steroids can effectively suppress lung inflammation, but they can also inhibit the body’s immune response and pathogen clearance [102]. Steroids were widely used during the SARS and MERS outbreaks, but their side effects also had a huge impact on patients’ health. In the current outbreak of SARS-CoV-2, the WHO on clinical management of severe acute respiratory infection recommend against the use of steroids unless specifically requested. If steroids are used, it is necessary to pay attention to monitor blood sugar, electrolytes during use, and may even appear symptoms of central excitation, such as insomnia, etc., can be symptomatic treatment. In general, steroid therapy is not recommended in the clinical treatment of SARS-CoV-2 [103]. Fibrinolytic enzyme levels and increased activity were found in blood tests of confirmed patients. In vitro experiments, it was found that the serine protease inhibitor TMPRSS2 could prevent novel coronavirus from entering cells, significantly improving the clinical prognosis of patients [104]. Vaccines are essential for preventing and treating infectious diseases. In addition to protecting healthy people from infection in an outbreak, vaccines can also help those infected rebuild their immune systems to control or cure the disease. Currently, many research teams are developing new coronavirus vaccines. The university of Pittsburgh study found a vaccine against SARS-Cov-2, which in animal studies has shown to make mice produce antibodies against SARS-Cov-2 and neutralize the virus [105]. In addition, some medical teams in China are using plasma from infected patients and traditional Chinese medicine treatment regimens. A study using convalescent plasma therapy found that in four of five infected patients, body temperature returned to normal after the infusion of convalescent plasma, and in three patients, mechanical ventilation was not required after 2 weeks. Although the number of cases in the study is small and the effect of convalescent plasma therapy still needs to be further verified, the effect is quite significant [57]. At present, there are successful cases of patients with new crowns all over the world, but it is difficult to carry out these protocols on a large scale due to the lack of systematic control analysis. Therefore, we look forward to the early development of effective treatments [106, 107].

## Prevention

In the current situation of rapid spread of the epidemic, and there is no special treatment, effective epidemic prevention measures, to contain the spread of the epidemic is crucial. At present, China’s “sealed” measures, restrictions on residents’ travel and traffic control have played a significant role in controlling the spread of the epidemic. Retrospective studies have found that traffic restrictions have significantly reduced the number of cases outside Hubei [108]. For China, which is celebrating the traditional “Spring Festival”, the traditional way of commemorating has been changed. All live parties have been cancelled, all scenic spots have been closed to tourists and crowds have been avoided by all means. Meanwhile, in an effort to prevent further spread of the virus, the Chinese government paid for the construction of two new hospitals in Wuhan in 10 days to treat the infected, and sent medical teams across the country to provide support. For countries outside China, the monitoring of passing tourists has been increased at airports, ports and other transportation arteries, mainly including temperature detection and symptom screening, to avoid the occurrence of imported cases. At present, China’s epidemic control is relatively stable, and its various measures have been copied by other countries [109]. Although the source and transmission route of SARS-CoV-2 are not clear enough, the main method is still to control the population flow, the early detection of cases, isolation of cases, and isolation of susceptible groups, do a good job of disinfection measures to prevent the further spread of the disease. In addition to significantly limiting the spread of the disease, the closure has also resulted in significant improvements in the city’s air quality [110]. In the event of a global pandemic, unnecessary travel should be avoided. In addition to limiting the movement of people and cutting off potential transmission, it is also important to protect individual residents from travel. In the early stage of the outbreak, due to the shutdown of various enterprises, insufficient production capacity of medical protective equipment such as masks and protective clothing, residents could not buy enough protective equipment, or even buy fake protective equipment, which could not play a protective role. A study of both surgical and cotton masks found that the virus appeared on the outside of the masks and did not completely prevent the virus from spreading, so hands must be disinfected when came into contact with the surface of the masks [36].

## Mental health

### The general public

To prevent outbreaks during epidemics, it is usually necessary to isolate high-risk groups and areas, restrict travel and movement of people, and try to avoid transmission between people. Since the outbreak, some measures have been taken all over the world, especially in China, where there has been a massive quarantine. For those who have experienced isolation, it is often an unpleasant experience. For uninfected people, during the period of isolation, they lose their freedom, lose their security, and tend to be impulsive and angry. During the period of isolation in various areas, there will often be conflicts between residents and epidemic prevention personnel. For those who are infected or may be infected, they will lose their freedom during the period of isolation, be separated from their families, and have insufficient understanding of their own illness. As a result, they are more likely to have psychological problems. In severe cases, they even give up their lives and commit suicide. Therefore, for large-scale isolation, it must be implemented on the basis of adequate measurement. In response to the outbreak, tens of thousands of infected people in China, where quarantine measures were first introduced, did not experience mass unrest. Instead, everyone was very cooperative with medical workers. People living in isolated homes and interacting with each other have been reported in the global media. Good mental attitude is also crucial for the recovery of the disease.

### The medical staff

As front-line medical workers, during the epidemic period, their workload significantly increased and their work risk also significantly increased. Therefore, their mental health needs more attention. High intensity medical work, lack of rest, but also need to undertake some patients do not understand the negative emotions, easy to focus on the work, poor emotional control and other uncomfortable performance. In order to timely understand the psychological health of medical staff, the anonymous psychological questionnaire can better help medical staff to solve psychological problems, help psychological counseling staff to better reduce pressure, enhance the information of medical staff, help them relieve psychological pressure, and better engage in medical work.

In a word, in order to minimize and avoid psychological and social problems caused by isolation, the most important thing is to shorten the isolation time as far as possible, the longer the isolation, the greater the impact. Scientific and reasonable isolation period may be able to make people more understanding, understanding, willing to accept, cooperate. In addition, the quarantined should actively provide more information to the quarantined during the quarantine period, and fully understand themselves’ health conditions and the status of the epidemic control, so that the quarantined can reduce fear. In addition, the quarantined personnel during the period of isolation, the loss of economic resources, the loss of basic security of life. Therefore, adequate material support is necessary for the quarantined people to meet their basic living needs and provide them with psychological comfort, making them more willing to believe that the national community can help them survive the epidemic [111–114].

## The role of new media

The emergence of new infectious diseases is a great challenge to global human health, public health and social development. In today’s society with developed network technology, the sharing of epidemic information plays an important role in the prevention and treatment of infectious diseases [115]. Under the strict epidemic prevention measures, the network and new media play an irreplaceable role of traditional media. But because cyberspace is a virtual world, there are real messages, but also false messages. Some malicious organizations or individuals use false information to cause social panic and hinder the prevention and control of the epidemic. At this point, the mainstream media needs to clarify in a timely and accurate manner to prevent the situation from getting worse.

The Internet and new media have played a crucial role in the outbreak. 1、Be the first to disclose the epidemic situation and play an early warning role. Network media workers as social current affairs scouts, with keen eyes, flexible ears and wise mind, constantly monitor the situation around, and can be the fastest speed to convey all kinds of news to the public. 2、Assisted traditional media to transmit epidemic information. After the outbreak of the epidemic, the public has an urgent need for information. New online media can disclose information timely, accurately and comprehensively, and some mainstream media can even invite the authorities to give professional explanations on the epidemic situation to meet the public’s information needs. 3、Actively guide public opinion, set up a correct value orientation. In a sudden outbreak, the general public will experience panic and negative behavior to some extent. In addition to timely transmission of information, mainstream network media can also carry out public opinion guidance, help the public correctly understand and analyze events, and cultivate the public’s psychological bearing capacity.

## Conclusions

SARS-CoV-2 is a highly transmissible virus that has caused a worldwide epidemic. At present, there is no specific treatment plan, which can only reduce the incidence of new cases by reducing the crowd gathering, virus elimination, personal protection and other work. In the nearly 4 months since the outbreak was discovered, more than a million people have been infected worldwide. Most countries around the world are testing their populations for potential cases, isolating them as nearly as possible and preventing the spread of the disease [116]. At first, it was thought that the virus would disappear in nature as temperatures rose, but the current global outbreak appears to be under control, with thousands of cases still being diagnosed in many countries every day. Some scholars predict that SARS-CoV-2 may persist in nature and may erupt repeatedly each winter [117]. In this outbreak, China performed very well and actively supported other countries to fight the epidemic after its own outbreak was under control. Facts have proved that China’s action to close down the city is correct. The rapid control of the epidemic cannot be achieved without the joint efforts of the government and the public. The whole country and all walks of life have paid a huge price. Most Chinese companies have gradually resumed production and most schools have opened. Wuhan, which had been “sealed”, has reopened. To prevent a repeat of the outbreak, the Chinese government has mandated that all returning workers and students be quarantined for 14 days, and that everyone in high-risk areas and occupations be tested for antibodies. Besides have obvious symptoms of infection, the crowd, there are a large number of asymptomatic infection, although these people themselves in good health, but will be coronavirus is positive for nucleic acid detection, and these asymptomatic infections can still will infect the others around you, the prevention and control of epidemic diseases bring huge challenges, only symptomatic infection control is not enough [118–121]. There are still many difficulties in preventing and controlling the epidemic. Thousands of people are diagnosed every day in the United States, Italy, Russia and other countries. As the currently has the largest number of the United States, confirmed its domestic unemployment increased, many small and medium-sized enterprise bankruptcy, the stock market turmoil, several historic fusing, part of the supply of medical resources, cost huge financial resources and forced the United States to increase the printing of dollars. This has also had a major impact on countries with dollar reserves, with some governments declaring bankruptcy [122]. One breath, one destiny, for all countries in the world, only by working together to fight the SARS-CoV-2 and help each other can we overcome the difficulties and restore prosperity as soon as possible.

## Data Availability

Not applicable.

## Abbreviations

SARS-CoV: Severe acute respiratory syndrome coronavirus
MERS-CoV: East respiratory syndrome coronavirus
SARS-CoV-2: Severe acute respiratory syndrome coronavirus2
WHO: The world health organization
ICTV: The international committee on taxonomy of viruses
ACE2: Angiotensin-converting enzyme 2
CDC: Centers for disease control and prevention
ESR: Erythrocyte sedimentation rate
GGO: Ground-glass opacity
